# Long-term outcomes of high-dose-rate brachytherapy and external beam radiotherapy without hormone therapy for high-risk localized prostate cancer

**DOI:** 10.1007/s11604-024-01621-4

**Published:** 2024-06-29

**Authors:** Nobuhiko Kamitani, Kenta Watanabe, Naoki Ikeda, Yujiro Kawata, Ryoji Tokiya, Takafumi Hayashi, Yoshiyuki Miyaji, Tsutomu Tamada, Kuniaki Katsui

**Affiliations:** 1 Department of Radiology, Kawasaki Medical School, 577 Matsushima, Kurashiki, Okayama 701-0192 Japan; 2 Department of Urology, Kawasaki Medical School, Kurashiki, Okayama Japan

**Keywords:** Prostate cancer, High-risk, Localized, Radiotherapy, High-dose-rate brachytherapy, Without hormone therapy

## Abstract

**Purpose:**

Until March 2018, patients with high-risk localized prostate cancer had been administered high-dose-rate brachytherapy (HDR-BT) combined with external beam radiotherapy (EBRT) without additional hormone therapy (HT) at our institution. In this study, we aimed to evaluate long-term outcomes of this treatment.

**Materials and methods:**

Patients with prostate cancer who received HDR-BT and EBRT between April 1997 and March 2021 and who were followed up for at least 6 months were included in the study. High-risk groups were classified into five levels according to the National Comprehensive Cancer Network guidelines. The EBRT and HDR-BT doses were 39–45 Gy/13–25 fractions. and 16.5–22 Gy/2–4 fractions, respectively. None of the patients received HT during initial treatment. The Kaplan–Meier method was used to estimate biochemical freedom from failure (bFFF), cause-specific survival (CSS), and overall survival (OS) rates. Biochemical failure was also determined.

**Results:**

Seventy-two patients were enrolled in the study, with a median follow-up of 91.9 months. The median age and initial prostate-specific antigen (iPSA) level were 71 years and 10.95 ng/mL, respectively. The median biologically effective dose for HDR-BT plus EBRT was 270.3 Gy. The 5- and 7-year bFFF, CSS, and OS rates were 85.2 and 74.2%, 100 and 100%, and 95.7 and 91.9%, respectively. Only the iPSA ≤ 20 group was associated with the higher bFFF rate. The 7-year bFFF rates in the groups with iPSA ≤ 20 and iPSA > 20 were 86.6 and 48.6%, respectively.

**Conclusion:**

HDR-BT plus EBRT without HT might be an alternative treatment option for patients with high-risk localized prostate cancer and iPSA levels ≤ 20. Further studies are required to validate the efficacy of this treatment strategy.

**Supplementary Information:**

The online version contains supplementary material available at 10.1007/s11604-024-01621-4.

## Introduction

Prostate cancer is a malignant tumor that is becoming increasingly prevalent and is the most common cancer in men worldwide [1, 2]. Local radical treatment with surgery or radiotherapy is the recommended standard of care for patients with localized prostate cancer. The National Comprehensive Cancer Network (NCCN) guidelines (version 4, 2023) recommend combining hormone therapy (HT) with local radiotherapy for high-risk patients. In randomized trials of adding the HT to external beam radiotherapy (EBRT), the group that received long-term HT experienced improved local control and cause-specific survival (CSS) rate [3–5]. Ishiyama et al. conducted a multicenter, retrospective study on prostate cancer treatment using EBRT and high-dose-rate brachytherapy (HDR-BT). They found that the group receiving HT had significantly better outcomes in terms of biochemical control and clinical disease-free survival (DFS), as well as overall survival (OS) rates, than of those who did not receive it [6]. Biochemical freedom from failure (bFFF) was considerably superior in the HT addition group in a study on low-dose-rate brachytherapy (LDR-BT) in patients with prostate cancer in the intermediate-risk group [7]. However, previous studies have indicated that the addition of HT does not improve treatment outcomes. The reports on EBRT with LDR-BT for prostate cancer showed that adding HT did not improve bFFF, CSS, or OS [8, 9]. Furthermore, HT has several adverse effects and may not be routinely added depending on the patient’s general condition and willingness to receive the treatment [10]. Therefore, identifying a subgroup of patients in the high-risk prostate cancer group who receive HDR-BT and require less HT would be beneficial; however, there is a lack of sufficient evidence regarding this distinction. Since 1997, our institution has performed HDR-BT combined with EBRT for localized prostate cancer. However, HT was not introduced until 2019, with the exception of neoadjuvant HT. In this retrospective study, we aimed to examine the long-term outcomes in patients diagnosed with localized high-risk prostate cancer who received HDR-BT combined with EBRT without HT. Moreover, we aimed to identify the subgroup of patients who achieved superior results without requiring HT.

## Materials and methods

### Selection of patients

This study was approved by the Ethics Committee of Kawasaki Medical School, Okayama, Japan (approval number 5329-1). All procedures were performed in accordance with the ethical standards set forth in the Declaration of Helsinki and its subsequent amendments. A notification on the website offered the opportunity to withdraw from this study. We examined patients with prostate cancer who received HDR-BT and EBRT at our institution between April 1, 1997, and March 31, 2021, and who were available for follow-up for at least 6 months after treatment. Treatment techniques were identical to those previously reported [11].

### Treatments

In this study, three-dimensional radiotherapy treatment planning in both HDR-BT and EBRT included the whole prostate as clinical target volume, but it excluded most of the seminal vesicles. HDR-BT was planned so that the minimum clinical target volume (CTV) dose was 95% of the prescribed dose. To comply with the organs at risk dose, the minimum CTV dose was allowed to be 90% of the prescribed dose. Urethra was allowed to be 120% of the prescribed dose and rectum 60% of the prescribed dose. EBRT was administered until March, 2009, using the four-field technique. Subsequently, three-dimensional conformal radiotherapy was initiated in April 2009 and intensity-modulated radiation therapy in April 2019. The HDR-BT and EBRT protocols were as follows: 16.5 Gy/3 fractions (Fr.) and 45 Gy/25 Fr. from April 1997 to March 1999, 22 Gy/4 Fr. and 45 Gy/25 Fr. from April 1999 to May 2000, 22 Gy/4 Fr. and 41.8 Gy/19 Fr. from June 2000 to December 2000, 24 Gy/4 Fr. and 36.8 Gy/16 Fr. from January 2001 to December 2006, and 20 Gy/2 Fr. (only 2, 18 Gy/2 Fr.) and 39 Gy/13 Fr. since January 2007. The treatment planning systems used for HDR-BT were PLATO (Nucletron, Veenendaal, Netherlands) and Oncentra version 3.3.86 (Nucletron) and version 4.5.3 (Nucletron). microSelectron version 2 (Nucletron) was used for treatment.

### Inclusion criteria

The patients were categorized into five groups based on the NCCN guidelines (version 4, 2022), and the high-risk groups were identified. The high-risk group was characterized according to the presence of any one of the following factors: cT3a, less than five cores with grade group 4 or 5, and initial prostate-specific antigen (iPSA) level > 20 ng/mL.

The minimum patient age was 20 years with a general condition (ECOG performance status) ranging from 0 to 2. Magnetic resonance imaging and computed tomography (CT) were performed in all patients, and no metastases were observed. HT was not administered during the initial treatment phase. Patients were eligible for inclusion in this study regardless of whether they underwent pelvic lymph node dissection (PLND) or sampling. Patients with insufficient pathology findings to diagnose the grade group before treatment were excluded from this study. Biochemical failure was determined based on the Phoenix definition [12].

### Statistical considerations

The bFFF, CSS, and OS rates were calculated. The bFFF is the biochemical relapse-free rate and is often used in prostate cancer analysis [7]. Grade 5 case was categorized as a prostate cancer-related fatality. The bFFF, CSS, and OS rates were analyzed based on the clinical T stage, biologically effective dose (BED), pathological grade, PLND, PSA level, and age. BED was calculated as follows: α/β = 1.5 [13]. Variables with p-values < 0.1 in the univariate analysis were further analyzed using the Cox proportional hazards model in a multivariate analysis. Statistical significance was defined as a p-value of < 0.05. Statistical analyses were performed using the SPSS version 20 software (SPSS Inc. Chicago, IL).

### Toxicity

The adverse events of genitourinary and gastrointestinal toxicities were assessed using the Common Terminology Criteria for Adverse Events version 5.0.

## Results

### Clinical characteristics

Patient characteristics are shown in Table 1. In all, 72 patients were treated, with a median observation period of 91.9 (range, 15.1–189.1) months. There was one case in which the patient did not receive the addition of HT after April 2019 at the patient’s own request. The median age and iPSA level were 71 (range, 52–81) years and 10.95 (range, 4.3–139.0) ng/mL, respectively. All patients had adenocarcinoma, and the grade groups were 1 for 14, 2 for 18, 3 for 8, 4 for 21, and 5 for 11 patients. In total, 9, 23, 13, 9, and 18 patients had T1c, T2a, T2b, T2c, and T3a stages, respectively. Forty-three patients underwent PLND. The four-field technique was performed in 42 patients, three-dimensional conformal radiotherapy in 29, and intensity-modulated radiation therapy in 1. Details of the HDR-BT combined with EBRT protocols are presented in Table 2. The median BED for HDR-BT combined with EBRT was 270.3 (range, 176.0–270.3) Gy. Biochemical failure was observed in 21 patients. The median time to biochemical failure was 60.03 (range, 7–171.43) months. Eleven patients died during the observation period. In all patients, HT was initiated after biochemical recurrence. The first site of clinical recurrence was bone metastasis in five patients, two were in the iPSA ≤ 20 group and three in iPSA > 20 group. There were two deaths due to prostate cancer, both in the high-dose group, one of which was classified as grade 5. There were nine deaths from other diseases with no metastases, and biochemical failure was seen in three.

**Table 1:** Patient characteristics

Number of patients	72
Age (years)	Median, 71 (range, 52–81)
iPSA (ng/mL)	Median, 10.95 (range, 4.3–139.0)
Grade group	
1/2/3/4/5	14/18/8/21/11
Clinical T stage	
1c/2a/2b/2c/3a	9/23/13/9/18
PLND	43

*iPSA* initial prostate-specific antigen, *PLND* pelvic lymph node dissection

**Table 2:** Details of the HDR-BT combined with EBRT protocols

EBRT	HDR-BT	BED (α/β = 1.5)	Number of patients
45.0 Gy/25 Fr	16.5 Gy/3 Fr	176.0 Gy	9
45.0 Gy/25 Fr	22.0 Gy/4 Fr	201.7 Gy	3
41.8 Gy/19 Fr	22.0 Gy/4 Fr	205.8 Gy	2
36.8 Gy/16 Fr	24.0 Gy/4 Fr	213.2 Gy	17
39.0 Gy/13 Fr	18.0 Gy/2 Fr	243.0 Gy	2
39.0 Gy/13 Fr	20.0 Gy/2 Fr	270.3 Gy	39

*EBRT* external beam radiotherapy, *HDR-BT* high-dose-rate brachytherapy, *BED* biologically effective dose, *Fr* fractions

### Oncological endpoints

**Fig. 1:**
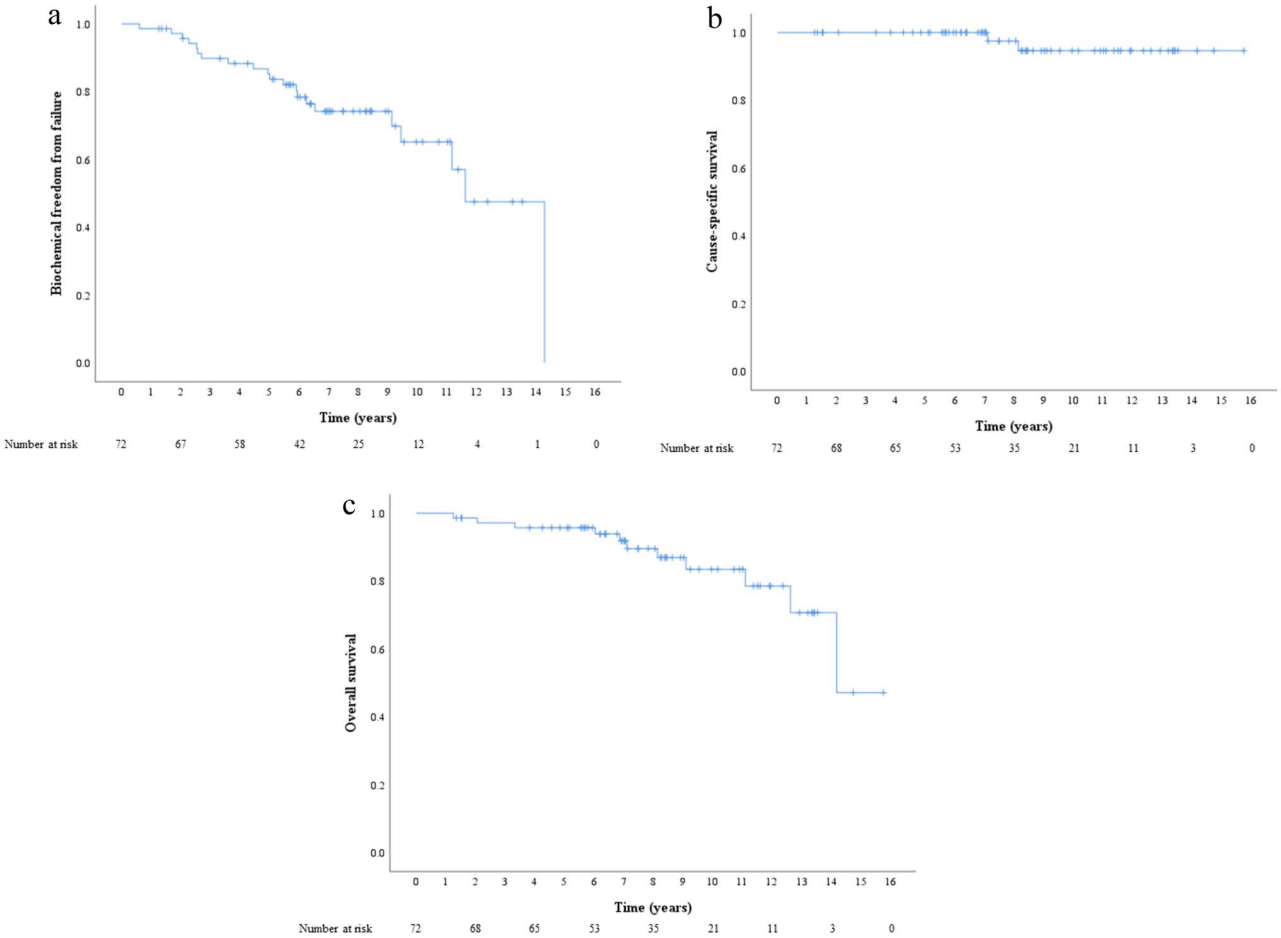
**a** Biochemical freedom from failure rate. The 5- and 7-year biochemical freedom from failure rates are 85.2 and 74.2%, respectively.** b** Cause-specific survival rate. The 5- and 7-year cause-specific survival rates are 100 and 100%, respectively. **c** Overall survival rate. The 5- and 7-year overall survival rates are 95.7 and 91.9%, respectively

**Fig. 2:**
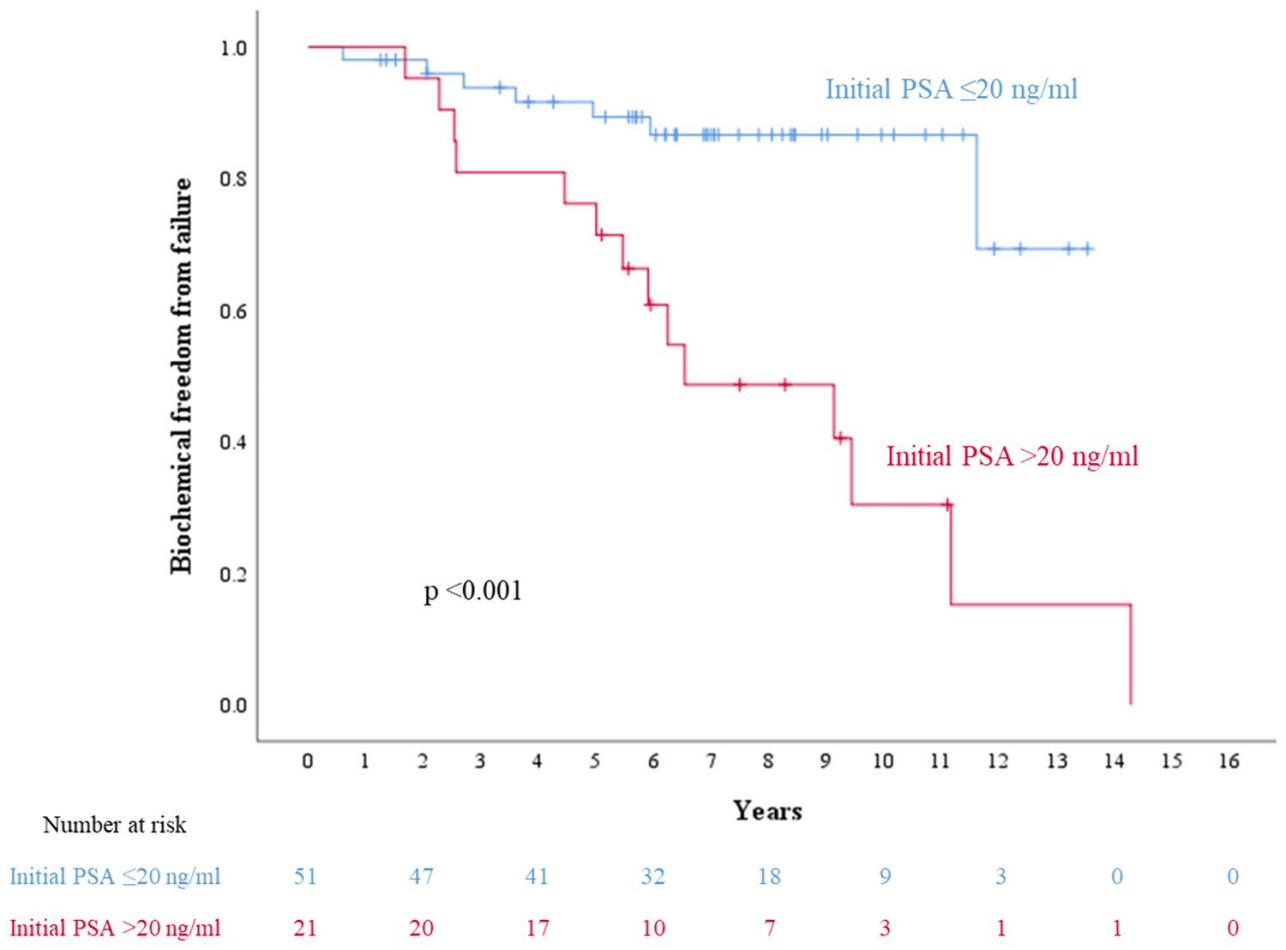
Biochemical freedom from failure of the iPSA ≤ 20 and iPSA > 20 groups. The 7-year biochemical freedom from failure rates in the iPSA ≤ 20 and iPSA > 20 groups are 86.6 and 48.6%, respectively. *iPSA* initial prostate-specific antigen

The 5- and 7-year bFFF rates were 85.2 and 74.2%, respectively (Fig. 1a). Univariate analysis was conducted for bFFF, CSS, and OS by dividing each factor into two groups. Age and BED were divided into ≥ 71 and < 71 median years and ≥ 270.3 and < 270.3 median Gy, respectively. iPSA, grade group, and clinical T stage were divided into high-risk group factors: > 20 ng/ml vs ≤ 20 ng/ml, ≥ 4 vs ≤ 3, and cT3a vs ≤ cT2. PLND was divided into two groups: with and without PLND. In the univariate analysis, the iPSA ≤ 20 and higher-grade groups were associated with the high bFFF group (p < 0.001 and p = 0.023, respectively). In the multivariate analysis, only the iPSA ≤ 20 group was associated with the high bFFF group (p = 0.024; Table 3). Details of the iPSA ≤ 20 and iPSA > 20 groups are shown in Supplemental Table 1. The 7-year bFFF rates in the groups with iPSA ≤ 20 and iPSA > 20 were 86.6 and 48.6%, respectively (Fig. 2). Of the five patients with distant metastasis after biochemical recurrence, two were in the iPSA ≤ 20 group and three in iPSA > 20 group. The 5- and 7-year CSS rates were 100 and 100%, respectively (Fig. 1b). In the univariate analysis, a lower BED was associated with a higher CSS rate (p = 0.043). The results of the CSS analysis are shown in Supplemental Table 2. The 5- and 7-year OS rates were 95.7 and 91.9%, respectively (Fig. 1c). Univariate analysis revealed no significant factors affecting OS. There were no significant differences in the CSS and OS between the groups with and without biochemical failure (p = 0.093 and 0.792, respectively).

**Table 3:** Univariate and multivariate analyses of factors associated with bFFF

				bFFF			
				Univariate analysis	Multivariate analysis		
		Number of patients	7-year bFFF (%)	p-value	p-value	Hazard ratio	95% CI
Age (years)	< 71	40	78.8	0.349	–		
	≥ 71	32	67.0				
iPSA (ng/ml)	≤ 20	51	86.6	< 0.001	0.024	0.234	0.067–0.825
	> 20	21	48.6				
Grade group	≤ 3	40	66.0	0.023	0.767	1.254	0.280–5.607
	≥ 4	32	85.4				
Clinical T stage	2	54	70.1	0.278	–		
	3a	18	88.5				
PLND	+	43	70.2	0.544	–		
	–	29	76.5				
BED (Gy)	< 270.3	34	68.3	0.192	–		
	≥ 270.3	38	79.0				

*bFFF* biochemical freedom from failure, *iPSA* initial prostate-specific antigen, *PLND* pelvic lymph node dissection, *BED* biologically effective dose

### Acute and late adverse events

No acute adverse events of grade 3 or higher were observed. Late adverse events included grade 3 urethral stricture in six patients, proctitis in one patient, and grade 5 bladder bleeding in one patient. Biochemical failure occurred 3 years and 6 months after treatment, and HT was initiated in a patient with grade 5 disease. Intermittent hematuria was observed 5 years after treatment, followed by refractory hematuria. Subsequently, he died 7 years and 1 month after the treatment.

## Discussion

Our study demonstrated that HDR-BT in combination with EBRT without additional HT resulted in 7-year bFFF, CSS, and OS rates of 74.2, 100, and 91.9%, respectively, indicating that iPSA was a significant prognostic factor for bFFF. The addition of HT to radiotherapy for high-risk patients with localized prostate cancer is standard of care and is intended to improve bFFF and survival [5, 14–16]. In these trials, radiotherapy was administered at doses as low as 70 Gy. Radiotherapy improves the rate of biochemical control in patients with prostate cancer by escalating the radiation dose, and the combination of brachytherapy and EBRT is also utilized to increase the dose to the prostate with minimal risk to neighboring organs. Ishiyama et al. conducted a large study of 3,424 patients treated with HDR-BT for prostate cancer and found that in the high-risk group, treatment of prostate cancer with the addition of HT resulted in better biochemical control, clinical DFS, and OS than that without additional HT [6]. Since 2019, when this paper was published, we have standardized the addition of HT to groups that are more advanced than the unfavorable intermediate-risk group at our institution.

The 8-year bFFF, CSS, and OS rates of 156 high-risk patients treated with HDR-BT in combination with EBRT without HT were 53.9, 95, and 76.1%, respectively [17]. Similarly, Prada et al. reported a 10-year bFFF rate of 74% [18]. For a study on LDR-BT without additional HT, Stone et al. reported that the high-risk group with GS ≥ 7 had a 5-year bFFF rate of 77.5% [19]. In a study of EBRT alone without additional HT, Krauss et al. reported 5-year bFFF and OS rates of 72.2 and 76.2%, respectively, in a high-risk group of 29 patients [17]. Our study results showed that the bFFF rate was comparable to those from other studies. The CSS and OS rates in our study were better than those reported previously. In our patients experiencing PSA recurrence, HT was immediately initiated in our study. If immediate administration of HT after PSA recurrence results in favorable CSS and OS, then a treatment strategy of HDR-BT in combination with EBRT, followed by deferral of HT, may be an alternative treatment for patients who do not wish to receive HT or are concerned about its adverse effects. Regarding CSS in our study, two patients in the higher BED group died of prostate cancer, resulting in a lower CSS in the higher BED group than in the lower BED group. In HDR-BT in combination with EBRT, there is a study reporting an improved CSS with higher doses, and our result differs from theirs [20]. Rationalizing our findings regarding prognostic factors for CSS is challenging. This detection may have incidentally occurred because of the limited number of cases or may have been affected by the inclusion of a grade 5 patient’s registration as a prostate cancer death.

In our study, the iPSA level was a significant prognostic factor for bFFF. Previous reports have shown improved outcomes in patients with iPSA levels ≤ 20 ng/mL. Krauss et al. reported that iPSA was a predictor of biochemical failure in all risk groups treated with EBRT alone or HDR-BT in combination with EBRT, with or without HT [17]. Martinez et al. also reported that iPSA and the Gleason score influenced biochemical failure in the treatment of HDR-BT in combination with EBRT and HT in high-risk groups [20]. Stone et al. administered LDR-BT in combination with EBRT without HT to patients with a Gleason score of ≥ 7 and found that the group with iPSA levels ≤ 20 ng/mL had improved bFFF [19]. Higher levels of PSA indicate a greater probability of detecting further lesions outside the prostate [21, 22]. Recent investigations using prostate-specific membrane antigen positron emission tomography (PSMA-PET) have also shown a positive correlation between PSA levels and the degree of extra-prostatic lesion accumulation [23, 24]. PSMA-PET has a superior detection rate compared with CT or bone scintigraphy for identifying extra-prostatic lesions in high-risk groups [25]. Additionally, Leeuwen et al. reported higher PSMA accumulation in patient post-prostatectomy with high PSA levels than in those not detected on CT or bone scintigraphy [26]. Biochemical recurrence was more prevalent in patients with iPSA levels > 20 ng/mL than in those with iPSA levels ≤ 20 ng/mL, and although PSMA-PET was not used in our study, it is reasonable to hypothesize the presence of extra-prostatic lesions. Our results showed a favorable 7-year bFFF rate of 86.6% in the iPSA ≤ 20 group, and HDR-BT in combination with EBRT without additional HT may be a promising alternative treatment for patients with iPSA levels ≤ 20 ng/mL.

In general, HT is associated with several adverse effects, such as sexual dysfunction, osteoporosis, hot flashes, worsening of metabolic disorders, fatigue, gynecomastia, reduction in penis and testicular size, weight gain, thinning hair, increased risk of cognitive decline, and exacerbation of cardiovascular disease and diabetes [27–31]. Yamazaki et al. found that prolonged HT for > 2 years might increase the possibility of mortality from non-prostate cancer causes [32]. A comparison of the outcomes of the short-term and long-term HT groups in the 10-year follow-up report of the DART trial demonstrated no statistically significant differences in biochemical DFS or OS in the high-risk group [33]. In the current age of high-dose radiation techniques, such as intensity-modulated radiotherapy and HDR-BT, the usefulness of HT may be less significant compared with that in the past, emphasizing the need for additional evidence.

Grade 5 bladder bleeding was observed in one patient. A study of 709 patients treated with radiotherapy suggested that the prevalence of hemorrhagic cystitis was not higher in patients who underwent brachytherapy [34]. Four patients (0.7%) had grade 5 hemorrhagic cystitis, but the study failed to specify the radiation technique that contributed to this event. In the grade 5 patient in our study, radiation doses were not markedly higher than those previously reported, and the underlying reason was not apparent, considering the patient background. Therefore, adequate informed consent and long-term follow-up are preferable.

Our study is limited by the small patient population at a single facility, the absence of standardized doses and fractions, and the potential benefit of excluding the very high-risk group classified by the NCCN. Ishiyama et al. reported a 10-year bFFF of 74.7% in their group without HT, compared with an improved 10-year bFFF of 83.0% in their group with HT in all risk groups [6]. Although the classification of high-risk groups in Yorozu et al.’s investigation differs from the one in our study, they reported a 7-year bFFF of 92% with the addition of HT in their study of LDR-BT [35]. In our study, the 7-year bFFF was 74.2%, a poor result compared to these reports with the addition of HT. Treatment without additional HT should be performed with informed consent. Nevertheless, our study is important because it demonstrated long-term outcomes, namely, a 7-year bFFF rate of 86.6% in the subgroup with iPSA ≤ 20.

In conclusion, we report the long-term outcomes of HDR-BT in combination with EBRT without additional HT for high-risk localized prostate cancer. Considering that the addition of HT is the standard of care for the treatment of high-risk localized prostate cancer, HDR-BT in combination with EBRT without the addition of HT might be an alternative treatment option for patients with iPSA ≤ 20 levels.

## Supplementary Information

Below is the link to the electronic supplementary material.

Supplementary file1 (DOCX 18 KB)
