# Supplementary material for: Long-term outcomes of high-dose-rate brachytherapy and external beam radiotherapy without hormone therapy for high-risk localized prostate cancer
Source: Jpn J Radiol. 2024 Jun 29;42(11):1322–9. doi: 10.1007/s11604-024-01621-4 (PMC11522092; doi:10.1007/s11604-024-01621-4)
Supplement: Supplementary file 1 — Supplementary file1 (DOCX 18 KB) [file 11604_2024_1621_MOESM1_ESM.docx]

Supplemental Table 1. Patient characteristics of the iPSA ≤20 ng/ml and >20 ng/ml groups

|  | iPSA ≤20 ng/ml | iPSA >20 ng/ml |
| --- | --- | --- |
| Number of patients | 51 | 21 |
| Age (years),  Median (range) | 69 (54–81) | 71 (52–79) |
| Grade group |  |  |
| 1/2/3/4/5 | 7/9/3//21/11 | 7/9/5/0/0 |
| Clinical T stage |  |  |
| 1c/2a/2b/2c/3a | 8/13/6/6/18 | 1/10/7/3/0 |
| PLND | 30 | 13 |
| BED≥270.3 Gy | 30 | 8 |

Abbreviations: iPSA, initial prostate-specific antigen; PLND, pelvic lymph node dissection, BED, biologically effective dose

Supplemental Table 2. Univariate and multivariate analyses of factors associated with CSS

|  |  |  |  | CSS |
| --- | --- | --- | --- | --- |
|  |  |  |  | Univariate analysis |
|  |  | Number of patients | 7-year CSS (%) | p-value |
| Age (years) | < 71 | 40 | 100 | 0.864 |
|  | ≥ 71 | 32 | 100 |  |
| iPSA (ng/ml) | ≤ 20 | 51 | 100 | 0.259 |
|  | > 20 | 21 | 100 |  |
| Grade group | ≤ 3 | 40 | 100 | 0.066 |
|  | ≥ 4 | 32 | 100 |  |
| Clinical T stage | 2 | 54 | 100 | 0.462 |
|  | 3a | 18 | 100 |  |
| PLND | + | 43 | 100 | 0.274 |
|  | - | 29 | 100 |  |
| BED (Gy) | < 270.3 | 34 | 100 | 0.043 |
|  | ≥ 270.3 | 38 | 100 |  |

Abbreviations: CSS, cause-specific survival; iPSA, initial prostate-specific antigen; PLND, pelvic lymph node dissection; BED, biologically effective dose
